# m^6^A modification is incorporated into bacterial mRNA without specific functional benefit

**DOI:** 10.1093/nar/gkaf425

**Published:** 2025-05-22

**Authors:** Klara Szydlo, Leonardo Santos, Thomas W Christian, Sunita Maharjan, Amir Dorsey, Isao Masuda, Jingxuan Jia, Yuan Wu, Weixin Tang, Ya-Ming Hou, Zoya Ignatova

**Affiliations:** Institute of Biochemistry and Molecular Biology, University of Hamburg, Hamburg 20146, Germany; Institute of Biochemistry and Molecular Biology, University of Hamburg, Hamburg 20146, Germany; Department of Biochemistry and Molecular Biology, Thomas Jefferson University, Philadelphia, PA 19107, United States; Department of Biochemistry and Molecular Biology, Thomas Jefferson University, Philadelphia, PA 19107, United States; Department of Biochemistry and Molecular Biology, Thomas Jefferson University, Philadelphia, PA 19107, United States; Department of Biochemistry and Molecular Biology, Thomas Jefferson University, Philadelphia, PA 19107, United States; Department of Chemistry, The University of Chicago, Chicago, IL 60637, United States; Institute for Biophysical Dynamics, The University of Chicago, Chicago, IL 60637, United States; Department of Chemistry, The University of Chicago, Chicago, IL 60637, United States; Institute for Biophysical Dynamics, The University of Chicago, Chicago, IL 60637, United States; Department of Chemistry, The University of Chicago, Chicago, IL 60637, United States; Institute for Biophysical Dynamics, The University of Chicago, Chicago, IL 60637, United States; Department of Biochemistry and Molecular Biology, Thomas Jefferson University, Philadelphia, PA 19107, United States; Institute of Biochemistry and Molecular Biology, University of Hamburg, Hamburg 20146, Germany

## Abstract

*N* ^6^-Methyladenosine (m^6^A), the most abundant modification in eukaryotic messenger RNAs (mRNAs), has also been found at a low level in bacterial mRNAs. However, enzyme(s) that introduce m^6^A modification on mRNAs in bacteria remain elusive. In this work, we combine deep-sequencing approaches that identify m^6^A sites with *in vitro* biochemical studies to identify putative m^6^A methyltransferases that would modify *Escherichia coli* mRNAs. We tested four uncharacterized candidates predicted to encode proteins with putative methyltransferase domains, whose deletion decreased the m^6^A level. However, *in vitro* analysis with the purified putative methyltransferases revealed that none of them installs m^6^A on mRNA. Exposure to heat and oxidative stress also changed the m^6^A level; however, we found no clear correlation between the m^6^A change and the specific stress. Considering two deep-sequencing approaches with different resolution, we found that m^6^A methylation on bacterial mRNAs is very low and appears randomly introduced. These results suggest that, in contrast to eukaryotes, the m^6^A modification in bacterial mRNA lacks a direct enzymatic recognition mechanism and has no clear biological function.

## Graphical Abstract

**Graphical Abstract:**
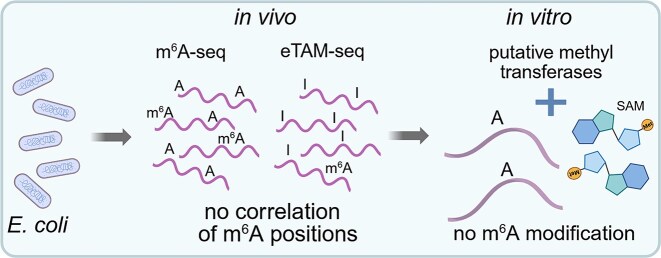

## Introduction

Modifications on RNAs are prevalent in all domains of life with a range of effects that broadly impact biology [1–3]. The most abundant one is *N*^6^-methyladenosine (m^6^A), which is present in transfer RNAs (tRNAs) and ribosomal RNAs (rRNAs) in all domains of life [4–7]. m^6^A is also the most common modification in eukaryotic messenger RNA (mRNA) and has been suggested to modulate a wide range of functions, including mRNA expression, stability, and metabolism [8–11], promoting alternative splicing [12, 13], and affecting nuclear mRNA export [14]. In eukaryotes, m^6^A is preferably installed on adenosines embedded within a DRACH motif (D = A/G/U; R = A/G; H = A/C/U) by a dedicated “writer” complex (e.g. the catalytic unit METTL3 assisted by METTL14 and the scaffolding WTAP protein), and it is removed by one of the m^6^A “erasers” (e.g. FTO or ALKBH5) [15–19]. However, in mammalian transcriptomes, only a small subset of adenosines (e.g. 0.2%–0.6% m^6^A/A) is methylated, with a strong positional enrichment toward the end of coding sequence (CDS) of transcripts and their 3′-untranslated regions (UTRs) [20–22], which is implicated as a positional specificity that is governed by the exclusion from the splice junctions [23–26].

The functional importance of m^6^A in eukaryotic mRNA raised the interest in exploring its importance in bacterial mRNAs. Notably, several m^6^A sites have been identified in the *Escherichia coli* transcriptome, based on antibody-recognition approach, followed by photo-crosslinking-assisted and high-throughput sequencing (PA-m^6^A-seq); however, m^6^A/A in bacteria is at a much lower level (0.02%–0.28%) relative to eukaryotic mRNA [27]. Unlike the uniform DRACH consensus motif in eukaryotes, in bacterial mRNA m^6^A has been found within multiple sequence motifs (e.g. GCCAU, UGCCAG, CAGAUC) [27]. Several m^6^A methyltransferases have been identified in *E. coli*, including the rRNA m^6^A methyltransferases encoded by *rlmF* and *rlmJ*, the rRNA dimethyltransferase encoded by *ksgA*, and the tRNA m^6^A methyltransferase encoded by the *yfiC* [28–30]; however, none of these modifies mRNAs [27]. Currently, no methyltransferase that modifies bacterial mRNAs is known. Without the identity of the m^6^A methyltransferase(s), it is difficult to establish the functional role of m^6^A in bacterial mRNAs.

Here, we sought to uncover the potential methyltransferase(s) that introduce m^6^A to bacterial mRNAs. Using *E. coli* as a model, we focused on four uncharacterized genes predicted to encode proteins with putative methyltransferase domains. While a deletion of each of these candidate genes decreased m^6^A levels in mRNA, *in vitro* analysis with purified proteins revealed no methylation on mRNA-derived oligonucleotides containing a consensus motif that was highly methylated in the m^6^A-seq data. To further elucidate the functional benefit for *E. coli*, we additionally characterized the m^6^A modification under two stress conditions. However, we found that m^6^A had no effect on the expression and stability of the modified transcripts. Two different sequencing approaches [i.e. an antibody-based m^6^A-seq [27, 31–34] and the new TadA-assisted *N*^6^-methyladenosine sequencing (eTAM-seq [22])] generated signals with little correlation. Together, our findings indicate that m^6^A modification on bacterial mRNAs lacks a specific enzyme-based installation mechanism and it appears to have no clear biological function.

## Materials and methods

### Homology search for putative m^6^A methyltransferases

The hidden Markov models (HMMs) of orthologous groups for bacterial methyltransferase domains were retrieved from the EggNOG database [35]. In total, seven groups were selected: COG0030 [rRNA (adenine-*N*^6^,*N*^6^)-dimethyltransferase activity]; COG0286 [site-specific DNA-methyltransferase (adenine-specific) activity]; COG0338 (D12 class *N*^6^-adenine-specific DNA methyltransferase); COG0500 (methyltransferase activity); COG2189 [belonging to the *N*^4^,*N*^6^-methyltransferase family]; COG2226 (methyltransferase); and COG3392 (D12 class *N*^6^-adenine-specific DNA methyltransferase). In *E. coli* as a Gram-negative model, all annotated genes were aligned to the HMMs from the orthologous groups using HMMER (v 3.1b2) [36]. The threshold of *e*-value <.01 was used to filter out low-confidence hits. The search resulted in 48 high-confidence hits, which we further screened for experimentally uncharacterized genes (Supplementary Table S1). Four of the uncharacterized hits (i.e. *yafE*, *yafS*, *yjhP*, and *ymfD*) are predicted to contain a putative methyltransferase domain. In separate BLAST searches using the NCBI and KEGG databases, we further confirmed that two of the four genes (*yafE* and *yihP*) were present in the Gram-positive bacterium *Bacillus subtilis*, indicating that they share homology across the bacterial domain.

### Strains and growth conditions

The *E. coli* MG1655 strain (F^−^ λ^−^ *ilvG^−^* *rfb*-50 *rph*-1) was used as a wild-type (WT) control strain. The *E. coli* K-12 KO strains ECK0210 (*yafE*-KO*)*, ECK0213 (*yafS*-KO), ECK4296 (*yjhP*-KO), and ECK1123 (*ymfD*-KO) were obtained from the Keio collection [37]. Of these, the *yafS*-KO strain overexpressing a plasmid-borne *yafS* was constructed as follows: the *yafS*-KO locus of the ECK0213 strain was transferred into *E. coli* MG1655 (DE3+) by P1 transduction. The *yafS* gene was synthetically built with optimized codons for expression in *E. coli* and was cloned into the BamHI and PstI restriction sites of pACYC-Duet vector under the control of the T7 promoter. The generated strain overexpressed *yafS* following induction with Isopropyl β-D-1-thiogalactopyranoside (IPTG). All strains were grown at 37°C in Luria–Bertani (LB) medium, but with addition of 50 μg/ml kanamycin (Sigma–Aldrich K1377) as a selection marker for the KO strains. For growth curve recordings, cultures were grown at 37°C in 96-well plates (Corning, Sigma–Aldrich) with orbital shaking in a multimode microplate reader (Tecan 426 Spark), or each in a 125-ml Erlenmeyer flask (20 ml medium) with steady shaking at 150 rpm. These were to ensure the most reproducible readouts [38]. Optical density at 600 nm was recorded at discrete intervals over 8–10 h.

To apply stress, overnight cultures of *E. coli* MG1655 of the WT strain (*yafS*-WT), the *yafS*-KO strain, and the two strains, each overexpressing *yafS*, were resuspended in fresh LB medium (Roth, #X968.4) and grown to mid-exponential phase at 37°C by rotation at 150 rpm. For heat stress (HS), cells were pelleted by centrifugation, and then resuspended in LB medium preheated to 47°C and incubated for 7 min at 47°C and orbital shaking at 150 rpm. For oxidative stress (OS), 1 mM paraquat (Merck, #36541) was added to the LB medium and cells were further incubated for 1.5 h at 37°C with rotation at 150 rpm. Thereafter, cells were immediately pelleted by centrifugation. Control cells unexposed to stress were further grown in LB medium at 37°C and orbital shaking at 150 rpm for the duration of the corresponding treatment in cells exposed to stress. Total RNA was extracted with TRIzol (Thermo Fisher, #15596026) according to the manufacturers’ instructions.

### Purification of YafS and YafE

*Escherichia coli yafE* and *yafS* genes were each amplified by polymerase chain reaction from the genomic DNA isolated from *E. coli* MG1655 and cloned into the restriction sites BamHI and XhoI of pET22b. The correct clone was confirmed by Sanger sequencing and transformed into *E. coli* BL21(DE3) for expression. Cells were grown to OD_600_ of 0.4–0.6 at 37°C, induced with 0.3 mM IPTG overnight at 16°C, and harvested. Cells were sonicated in lysis buffer (25 mM Tris–HCl, pH 7.4, 10 mM Mg(OAc)_2_, 0.1 mM 1,4-dithiothreitol (DTT), 0.1 mM ethylenediaminetetracetic acid (EDTA), and 5% glycerol) supplemented with a tablet of protease inhibitors (Thermo, #A32953). Cell lysates were passed through a His-Trap HP column (Cytiva, #17524802). The column was washed with five volumes of the lysis buffer, containing 10 mM imidazole; where necessary, washes were continued until stable baseline at OD_280_ was reached. YafE or YafS protein was eluted by a gradient of imidazole from 10 to 300 mM over 30 min. Protein-containing fractions, as analyzed on 15% sodium dodecyl sulfate–polyacrylamide gel electrophoresis, were pooled and concentrated in 50 mM 4-(2-hydroxyethyl)-1-piperazineethanesulfonic acid (HEPES), pH 7.5, 20 mM Mg(OAc)_2_, 300 mM NaCl, 0.2 mM DTT, and 0.2 mM EDTA using a Vivaspin concentrator (Sartorius, #VS15T01). The concentrated YafE and YafS proteins exhibited 90% or 70% homogeneity, respectively. Each protein was mixed with an equal volume of 80% glycerol and stored at –20°C.

For assays using cell lysates, each cell lysate was prepared as described [39]. Briefly, cells were harvested at OD_600_ of 0.4–0.6, resuspended in a lysis buffer [10 mM Tris–HCl, pH 7.5, 30 mM KCl, 10 mM MgCl_2_, 1 mM DTT, 1 mM PMSF, and one tablet of protease inhibitors (Pierce, #PIA32953)], and lysed in a sonication bath at the maximum power for three bursts of 30 s each. After cell debris was removed by centrifugation (12,225 × *g*, 30 min, 4°C), ribosomes were removed by a second spin at 500,000 × *g* in an S100-AT4 rotor in an MTX-150 centrifuge for 2 h at 4°C. The cleared supernatant of the lysate was recovered from the second spin and incubated with a DEAE Sepharose resin in the lysis buffer for 1 h to bind proteins with affinity to DNA or RNA. The DEAE Sepharose resin was washed to remove the unbound fraction, while the bound fraction was eluted with an elution buffer [20 mM Tris–HCl, pH 7.4, containing 10 mM Mg(OAc)_2_, 0.1 mM DTT, 0.1 mM EDTA, 100 mM imidazole, and 5% glycerol], and protein concentration was determined. In each assay with a cell lysate, each cellular protein was assumed an average molecular weight of 50 kDa, and the methyltransferase of interest was assumed at 1% of total cellular proteins, which was tested at 150 nM against an RNA substrate.

### *In vitro* m^6^A methylation assays

Two RNA oligonucleotides (5′-AACUUCGCACUGCC**A**GCA GUAAACUGCGUCGGUACUGACUCC-3′) that differ in the methylation status of the A nucleotide (bold), i.e. m^6^A modified (designated as the m^6^A-mRNA) and unmodified (designated as A-mRNA), were purchased from Dharmacon (Horizon Discovery, GE Healthcare). The RNA oligonucleotide sequence is within the coding region of *E. coli fbaA* gene (for fructose-bisphosphate aldolase class 2), and the highlighted A nucleotide was with the highest methylation frequency in our m^6^A-seq data set. Notably, this A nucleotide is in the identified m^6^A motif (UGCCAG) in *E. coli* [27]. The RNA oligonucleotides were designed to retain the predicted mRNA secondary structure based on M-fold. Each mRNA oligonucleotide was heat-denatured and refolded in 10 mM Tris–HCl, pH 8.0, and 10 mM MgCl_2_ for 15 min prior to assay.

Each *in vitro* m^6^A methylation reaction was performed in the methylation buffer (50 mM Tris, pH 8.0, 5 mM MgCl_2_, 1 mM DTT, 50 mM NaCl, and 0.1 mM EDTA), which was tested to support a variety of RNA methyltransferases, including *E. coli* TrmD and human Trm5 [40], the human METTL3–METTL14 [41], and the *E. coli* TrmH [42]. The components of the reaction, mRNA oligonucleotide (16 μM), [^3^H]-*S*-adenosyl methionine (SAM) (50 μM), and cell lysates or recombinant YafE or YafS (4 μM), were mixed at 37°C in the presence of the Ribolock RNase inhibitors (Thermo Fisher, #EO0381) at 40 units per reaction. The transfer of the [^3^H]-methyl group from SAM onto the RNA substrate was measured by scintillation counting of the reaction precipitated on acid filter pads, which were then converted to picomoles. Aliquots of the reaction were sampled over time (15–45 min), precipitated on filter pads in trichloroacetic acid (TCA, w/v, 5%), washed with ethanol (95%) twice and ether once, air dried, and the radioactivity measured by an LS6000 scintillation counter (Beckman). A negative control reaction without YafE or YafS was run in parallel to determine the background counts, which were verified in a Michaelis–Menten analysis of the positive-control reaction—the *N*^1^ methylation of G37 in tRNA [43]. The background counts were then subtracted from the counts of an enzyme-catalyzed methyl transfer reaction and converted to picomoles, using the specific activity of [^3^H]-SAM after correction for the signal quenching factor by filter pads. In each time course of a methylation assay, the picomoles of the negative control were set to 0 and the background-subtracted picomoles in each time point were plotted. The fraction of the methylated RNA was then calculated relative to the input RNA substrate in each assay.

### SAM binding assays

These assays were performed to measure the thermodynamic equilibrium binding affinity of each recombinant protein to SAM as previously described [43]. Briefly, the binding of SAM to YafE or to YafS was determined by measuring the quenching of the intrinsic tryptophan fluorescence of each protein. Purified recombinant *E. coli* YafE or YafS (0.5 μM) was separately titrated with SAM (at room temperature) in a binding reaction. With each purified protein, SAM was titrated in the range of 0–20.6 μM, and the fluorescence of the protein was measured with excitation at 288 nm and emission at 330 nm. Each emission measurement was baseline corrected with the buffer (100 mM Tris–HCl, pH 8.0, 100 mM KCl, 0.1 mM EDTA, 6 mM MgCl_2_, and 4 mM DTT). Fluorescence changes were fit to a saturable hyperbolic binding equation to determine the *K*_d_ of binding. The reported *K*_d_ of each protein is an average value of three independent binding titration experiments.

### m^6^A sequencing

#### Experimental design

Cells from the mid-exponential phase grown at permissive conditions or exposed to OS and HS (as described earlier) were harvested by centrifugation and total RNA was extracted using TRIzol. A minimum of 400 μg total RNA was isolated from each sample and fragmented using RNA fragmentation reagents (Ambion, #AM8740). An aliquot of the fragmented RNA from each sample (5 μg) was saved for RNA-seq as input control. The remaining RNA was incubated with 2.5 μg of anti-m^6^A antibodies from mouse (Synaptic Systems, #202003) and with 10 μg of anti-m^6^A antibody from rabbit (Merck Milipore, #ABE572) in immunoprecipitation (IP) buffer (10 mM Tris–HCl, pH 7.4, 150 mM NaCl, 0.1% Igepal) for 3 h at 4°C. Thereafter, the reaction was incubated with Protein A/G magnetic beads (Thermo Fisher, #88802) for 3 h at 4°C. The m^6^A RNA bound to the beads was eluted using hot acid phenol as previously described [31]. ERCC RNA Spike-in mix (Thermo Fisher, #4456750) was added to the input samples, and rRNA depletion was carried out using *E. coli* RiboPOOL kit (siTOOLs). The rRNA-depleted RNA was then used for complementary DNA library construction and sequencing as described previously [44]. The libraries were sequenced on Illumina HiSeq4000 platform. All samples were sequenced with two biological replicates, except the KO samples, which was sequenced in a single replicate.

#### m^6^A sequencing analysis and identification of m^6^A sites

The sequenced reads were trimmed using fastx-toolkit with a quality threshold of 20. The adapter sequences were removed using cutadapt with a minimal overlap of one nucleotide [45]. The reads were aligned to *E. coli* rRNA sequences and the unmapped were kept. The processed reads were aligned to the reference genome using Bowtie algorithm [46] allowing only uniquely mapped reads with a maximum of one mismatch (parameters: -l 16 -n 1 -e 50 -m 1 --strata --best -y). The *E. coli* K-12 sub-strain MG1655 (ENSEMBL: GCA_000005845) was used as the reference genome for the mapping. Bedtools was used to count the number of reads mapped to each single gene annotated [47]. Final mapped reads were normalized as reads per kilobase per million mapped reads (RPKM).

The mRNA m^6^A modification sites were identified as previously described [48]. Briefly, for each individual gene a sliding window of 50 nucleotides (25 nt as a sliding step) was used to calculate the peak over median (POM), which is the ratio of the mean coverage within the sliding window over the median coverage across the transcript. Regions with coverage <10 and a POM score <3 were discarded. We calculated separately the POM for input and IP samples. Shared regions between input and IP were also discarded. Subsequently, we calculated the peak over input (POI), which is the ratio of the POM score for each region in the IP sample over the input sample. Peaks with POI score >3 were considered for the downstream analysis. The peaks identified for each of the biological replicates were merged into one set of unique peaks for each condition. Finally, we selected the m^6^A peaks containing one of the previously described m^6^A methylation motifs, e.g. GCCAU, UGCCAG, and CAGAUC [27]. These motifs were identified using HOMER algorithm [49]. The metagene plots were generated by binning each transcript into 10 bins and calculating the ratio of the detected m^6^A-modified motifs over the total putative motifs identified in each bin. Mann–Whitney test was used to determine the statistical significance between m^6^A distribution pattern between permissive growth and following stress exposure.

For the comparison with eTAM signals, we used unfiltered m^6^A peaks. Namely, all peaks with POI > 3 from both replicates were merged into one set and no further selection of m^6^A sites within previously suggested motifs [27] was performed.

### eTAM sequencing

#### Experimental design

TadA8.20 was overexpressed and purified following a previously published protocol [22] using expression plasmid Addgene #194702. Fifty nanograms of rRNA-depleted *E. coli* RNA was subjected to the eTAM-seq workflow. Briefly, RNA was deaminated with 200 pmol of TadA8.20 in 20 μl of 1× deamination buffer (50 mM Tris, 25 mM KCl, 2.5 mM MgCl_2_, 2 mM DTT, and 10% (v/v) glycerol; pH 7.5) supplemented with 10% (v:v) SUPERase•In RNase Inhibitor at 53°C for 1 h. The treated RNA was purified using RNA Clean & Concentrator kits (Zymo, #R1016), and the deamination reaction was repeated twice at 44°C. TadA8.20-treated RNA was built into libraries using the NEBNext Ultra Directional RNA Library Prep Kit for Illumina (New England Biolabs, #E7760L), following the manufacturer’s protocol (section 4 for purified mRNA, skipping fragmentation). The input library was constructed using the same kit with 50 ng RNA as input. Libraries were quantified using the KAPA Library Quantification Kit (KAPA Biosystems, #KK4824) and sequenced on a NextSeq 2000 instrument (R1 25 cycles, R2 97 cycles, Illumina, #20024906). The eTAM sequencing was performed in two independent biological replicates.

#### eTAM-sequencing analysis

Sequenced reads were submitted to the eTAM-seq analysis pipeline, available in the GitHub repository (https://github.com/shunliubio/eTAM-seq_workflow). Briefly, 5′ and 3′ reads were trimmed from the adapters using cutadapt with the default parameters from the pipeline. Next, rRNA reads were depleted from the dataset following mapping to the *E. coli* rRNA reference sequence with hisat3n aligner. The remaining reads were mapped to the *E. coli* K-12 sub-strain MG1655 (ENSEMBL: GCA_000005845.2) reference genome.

Typically, to select for genuine m^6^A sites, eTAM-seq incorporates a parallel treatment with the human m^6^A eraser FTO to enzymatically deaminate methylated adenosines [50]. The application of this step in *E. coli* is not possible; TadA8.20 is purified from overexpressing bacterial cultures and the final preparation is contaminated with low amount, yet detectable in sequencing, *E. coli* RNA. FTO-treated samples are particularly susceptible to the contamination due to FTO-induced RNA degradation. Thus, we directly used persistent A signals detected in eTAM-seq in downstream analysis, which might be either m^6^A or structured adenosines. The two ribosomal m^6^A sites, namely positions 1618 and 2030 in 23S rRNA, were robustly detected and served as an internal control. By calling all A sites with at least 50% persistent A signals (depth filter 20), we detected 1690 and 1703 sites in each of the two biological replicates. The majority of them (1014) overlapped.

## Results and discussion

### Four putative uncharacterized methyltransferases do not confer m^6^A activity on mRNA

In analogy with eukaryotic m^6^A modification machinery, we hypothesized that the m^6^A modification should be installed on *E. coli* mRNAs by an m^6^A writer (or a writer complex) with a methyltransferase activity. No prokaryotic homologues exist for the eukaryotic METTL3 and METTL14 [27]. We thus conducted a homology search using known methyltransferase domains of bacteria and obtained 48 hits (Supplementary Table S1). Many of these had already been characterized as methyltransferases that act on rRNA, DNA, or tRNA, or with functions not related to methylation of nucleic acids (e.g. *speE*, a methyltransferase in polyamine biosynthesis). The four uncharacterized hits (i.e. *yafE*, *yafS*, *yjhP*, and *ymfD*) were predicted to have a putative methyltransferase activity (Supplementary Table S1). To identify hits with the most meaningful homology across the bacterial domain, we performed two additional BLAST searches against the Gram-positive bacterium *B. subtilis* using the NCBI and KEGG databases. The search suggested that *yafE* is related to a methyltransferase in quinone biosynthesis, and that *yjhP* is related to a sterol-*C*-methyltransferase or possibly 3-demethyl-ubiquinone-9,3-methyltransferase. We detected no hits for *yafS* and *ymfD* in *B. subtilis*. Since two of these four genes were implicated into funneling metabolites into RNA methylation, we considered all four genes to investigate their roles in m^6^A methylation of *E. coli* mRNAs.

We obtained the KO strains Δ*yafE* (ECK0210), Δ*yafS* (ECK0213), Δ*yjhP* (ECK4296), and Δ*ymfD* (ECK1123) from the *E. coli* Keio collection [37]. We next assessed the effect of the loss of each gene on *E. coli* growth, since growth aberrations have been observed following a KO of other methyltransferases, e.g. rRNA methyltransferase *rlmE* [51]. However, none of the KO strains negatively affected *E. coli* growth (Supplementary Fig. S1A). As exemplified with *yafS*, a plasmid-borne supplementation of the deleted gene in the WT or KO strain did not change the growth profile (Supplementary Fig. S1B).

**Figure 1.**
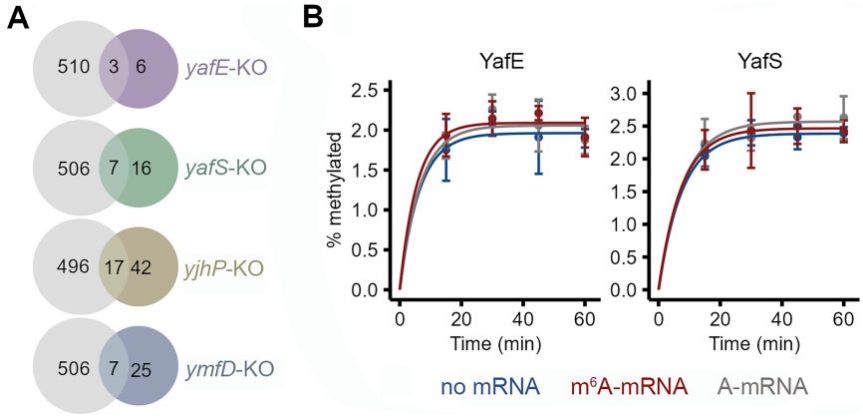
The m^6^A activity of four putative *E. coli* methyltransferase candidates. (**A**) Overlap of methylated transcripts with at least one m^6^A site between the *E. coli* MG1655 WT strain (gray circles) and each of the four KO strains (colored circles). In each comparison, the WT strain was sequenced in two independent biological replicates, and data were pooled into a metaset, while each KO strain was sequenced with one biological sample. (**B**) Time course of *in vitro* methylation assay with purified YafE and YafS on the A-mRNA substrate compared to m^6^A-mRNA, and to no-mRNA reaction . In each assay, 16 μM purified YafE or YafS and 4 μM mRNA substrate were used. The percentage of methylation was calculated by subtracting the counts of a no-enzyme control from the counts of mRNA-associated [^3^H]-SAM counts and reported as percent of pmoles. The no-enzyme control reaction was set to 0 for each time course. The reaction lacking mRNA substrate (no mRNA) served as a negative control, which was run in parallel to all analysis. Data are means ± SD (*n* = 3 independent biological replicates) and were fit to a single exponential equation based on searches for the best fit.

To assess the effect of the KO of these four genes on the m^6^A modification levels of mRNA, we carried out m^6^A-seq on each KO strain and compared it to the *E. coli* WT MG1655 strain. We reasoned that if any of these putative methyltransferases would be an m^6^A writer by itself, KO of the gene should theoretically eliminate all m^6^A sites on the transcripts. We used the antibody-based m^6^A-seq approach [27, 31–33]. In peak calling, the identified m^6^A peaks containing one of the previously described m^6^A methylation motifs, e.g. GCCAU, UGCCAG, and CAGAUC [27], were considered truly positive. As the antibody-based approach lacks nucleotide precision, in the further analysis we considered all transcripts with at least one m^6^A peak, and not the precise m^6^A peak positions. In all KO strains, we found a significant reduction of the methylated transcripts (Table 1), with Δ*yafE* exhibiting the largest decrease, followed by Δ*yafS* (Fig. 1A). Notably, among transcripts that remained persistently methylated in the KO strains, the majority of them were unique to each KO strain, with marginal overlap with the WT strain (Fig. 1A) or between KO strains (Supplementary Fig. S2). We expected that, if any of the putative methyltransferase was the single enzyme responsible for the m^6^A modification, the major difference between the KO and the WT would be a loss of m^6^A-methylated transcripts without appearance of any newly methylated transcripts. Thus, the unique m^6^A-modified mRNAs that appear in all KO strains suggests that none of the four genes alone was solely responsible for m^6^A installation and/or that other mechanisms might account for the mRNA methylation.

**Table 1.** Decrease in methylation upon KO of putative methyltransferase genes

Strain	Number of m^6^A peaks	Number of m^6^A-modified transcripts
WT MG1655	602	509
*yafE*-KO	9	9
*yafS-*KO	23	23
*yjhP*-KO	63	59
*ymfD*-KO	32	32

Number of m^6^A peaks and genes determined following m^6^A-seq in different strains is shown in each case.

### Purified putative methyltransferases have no m^6^A-mRNA methylation activity

Deletion of *yafE* and *yafS* caused the largest decrease of methylated transcripts, i.e. leaving in each KO strain 9 and 23 m^6^A peaks, respectively (Table 1 and Fig. 1A). Thus, we next tested the m^6^A activity in lysates from WT cells and each *yafE* or *yafS* KO strain using an RNA oligonucleotide as a substrate. The sequence of the RNA oligonucleotide was selected from the m^6^A-seq dataset as the most frequently methylated in both biological replicates of the WT cells. In addition, this position corresponds to a previously identified mRNA stretch as methylated that falls within the proposed UGCCAG methylation motif [27]. We designated the unmethylated RNA oligonucleotide as A-mRNA. As a control, we also synthesized the corresponding oligonucleotide with methylated adenosine and designated it as m^6^A-mRNA. In WT lysates, we did not detect any m^6^A modification on the A-mRNA substrate above the background (Supplementary Fig. S3). To validate that the lysate was competent to perform methylation of RNA, we showed that it was active in performing the *N*^1^ methylation of guanosine at position 37 of tRNA (m^1^G37), indicating that the dedicated enzyme TrmD was catalytically active in the lysate (Supplementary Fig. S3).

The lack of the m^6^A modification on the oligonucleotide might be due to limited levels of the m^6^A methyltransferase in the lysates. To address this possibility, we purified YafE and YafS from a strain overexpressing each protein and tested the methylation activity *in vitro*. With the purified YafE or YafS being in a four-fold molar excess over the A-mRNA substrate, we did not observe any methylation activity above the background (Fig. 1B). The calculated fraction of the methylated A-mRNA was very low (∼2%); the change in the signal was essentially the same as that of the m^6^A-mRNA substrate or of the reaction without the mRNA (Fig. 1B). Together, we found no detectable m^6^A methyltransferase activity of the YafE and YafS proteins, either in cell lysates or in their purified form. We chose reaction condition compatible with a wide range of RNA methyltransferase enzymes across species, including *E. coli* TrmD, human Trm5 [40], the human METTL3-METTL14 complex [41], and the *E. coli* TrmH enzyme (catalyzing 2′-*O*-methylation of G18) [42]. Although we cannot entirely exclude the possibility that suboptimal reaction parameters contributed to the lack of detectable activity, this negative result is consistent with our other data, including transcriptome-wide m^6^A-seq analyses.

Although we did not observe any m^6^A-mRNA methylation activity of YafE or YafS (Fig. 1B), we further tested whether either of them may have an affinity to bind the methyl donor SAM and act as SAM-dependent methyltransferase. We used our previously developed fluorescence titration assay, which reports on SAM-binding activity by monitoring changes of the intrinsic tryptophan fluorescence of the target protein [43]. With this assay, we observed no change of the intrinsic fluorescence of YafE, suggesting no SAM binding (Supplementary Fig. S4A). In contrast, YafS exhibited a progressive decrease of the fluorescence as a function of the SAM concentration, starting from the reference level at 100% and plateaued at 85%. In the control reaction lacking SAM, the YafS fluorescence plateaued at 95%. Fitting the titration data to a thermodynamic hyperbolic binding equation revealed an equilibrium dissociation constant *K*_d_ of 0.68 ± 0.1 μM (Supplementary Fig. S4B). This *K*_d_ is similar to that of the tRNA-methyltransferase TrmD upon binding of SAM (*K*_d_ = 1.0 μM) [43]. Together, these results indicate that although YafS has the capacity to bind SAM similarly to other methyltransferases, it did not exhibit m^6^A activity on the A-mRNA substrate.

It should be noted that we did not test whether YafS would gain on methylation activity in complex with YafE or with any of the other putative methyltransferases, based on the notion that all known *E. coli* methyltransferases, acting on tRNA [52] or rRNAs [51], are single enzymes. An activation of YafS as a complex with YafE seems unlikely, as each of them alone had no activity on the A-mRNA substrate. Furthermore, the growth curves of the WT and *yafS-*KO were largely similar, and remained similar even upon overexpression of a plasmid-borne *yafS* (Supplementary Fig. S1B), supporting the notion that YafS alone has no strong phenotypic effect. In contrast, knockdown of the eukaryotic METTL13 or METTL14 has a clear negative effect in various cell types [53–57]. Knockdown of *E. coli* methyltransferases, which have a well-defined role in physiology, also alters growth phenotypes. For example, the knockdown of the tRNA methyltransferase gene *trmD* severely impairs cell growth [58], and this impairment is rescued by TrmD overexpression [59]. These comparisons further indicate that YafS alone likely has no physiological relevance for *E. coli* under permissive growth conditions.

To test whether YafE or YafS could methylate single nucleotides or nucleosides, which then can be incorporated into nascent mRNA transcripts, we incubated each purified protein with adenosine, AMP, or ATP. We did not find any *N*^6^-methylated products by direct liquid chromatography-tandem mass spectrometry (LC–MS/MS) analysis, suggesting that the putative methyltransferase domain of YafE or YafS cannot catalyze methylation of single nucleotides or nucleosides.

### m^6^A modification levels decrease stochastically following stress exposure

**Figure 2.**
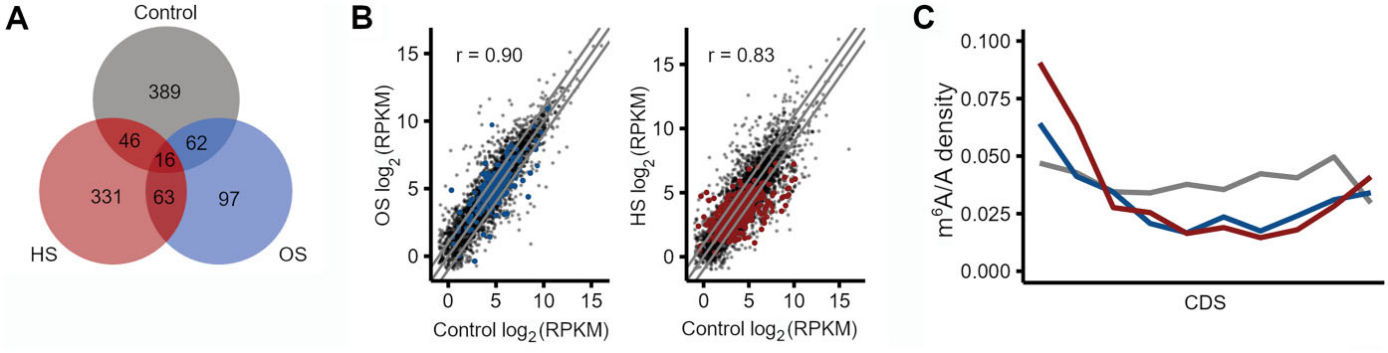
Alterations of methylation following environmental stress exposure. (**A**)Overlap of m^6^A-modified transcripts with at least one m^6^A site in the control cells with those in cells exposed to OS or HS. (**B**) Comparison of transcripts expression in control cells (*x*-axis) with those expressed in cells exposed to OS (*y*-axis, left plot) or HS (*y*-axis, right plot) determined from RNA-seq. Colored dots represent methylated transcripts under OS (left plot) or HS (right plot), respectively. *r*, Pearson correlation coefficient; lines parallel to the diagonal delineate the threshold [log_2_(FC) = ±1] of differentially expressed transcripts. (**C**) Metagene profiles of m^6^A distribution along the entire CDS length (*x*-axis) of methylated transcripts at permissive control growth (gray), or exposed to OS (blue) or HS (red). Detected m^6^A sites are expressed as a ratio of total m^6^A sites and following aggregation across all mRNAs are represented as density distributions. To normalize CDS lengths, each CDS was segmented in equal-sized bins, which were then merged to generate uniformly scaled CDS lengths. Changes in m^6^A distribution following stress are statistically insignificant. m^6^A-seq and RNA-seq (A–C) were performed on two independent biological replicates for each condition and both replicates were merged into one metaset.

We next hypothesized that the methylation might be important under stress. We thus assessed the dynamics of the m^6^A modification in cells exposed to HS or OS. Although we did not identify any m^6^A writer so far, we reasoned that, similar to eukaryotes, an enzyme-targeted m^6^A modification could be sensitive to stress [31, 60]. We isolated total RNA of *E. coli* cells exposed to HS and OS, and assessed the global m^6^A levels using antibody-based m^6^A-seq. At permissive growth conditions, within CDSs, we identified 602 m^6^A sites within the previously identified consensus motifs that were spread across 513 transcripts (Fig. 2A). Following exposure to stress, the number of the m^6^A-modified transcripts decreased, corroborating previously detected decrease of m^6^A in *Pseudomonas aeruginosa* upon exposure to HS [27]. We observed a much larger decrease in OS; i.e. 248 m^6^A peaks within 238 transcripts were identified in OS as compared to 513 m^6^A peaks on 456 transcripts in HS (Fig. 2A). However, the identity of the m^6^A-modified transcripts in cells exposed to each stress condition was unique with little overlap between each stress type and control (Fig. 2A). This would indicate that transcripts that are m^6^A-modified under permissive growth (control) would be specifically degraded in stress, and/or m^6^A would be *de novo* installed under stress. We compared the transcript expression levels from the RNA-seq experiment accompanying the m^6^A-seq. The strong correlation of the transcript expression levels between control cells and cells exposed to stress indicated a negligible contribution of mRNA degradation in stress (Fig. 2B). Thus, the observed decrease in m^6^A methylation levels under stress was not due to mRNA degradation. The lack of a specific enzyme dedicated to m^6^A installation rather suggests that completely different transcript sets might have been methylated from the onset of cell growth prior to stress exposure.

Next, to establish any role of m^6^A in stress response, we tested whether the distribution of m^6^A modification along the CDS changed in response to each type of stress, which would indicate a direct role of m^6^A in stress. Following each stress exposure, we did not observe m^6^A alterations along the CDSs that reached statistical significance (Fig. 2C). Although global levels of m^6^A methylation decreased following stress exposure (Fig. 2A), we did not detect transcript-specific changes. Compared to wild type, the initial m^6^A increase following HS or OS was compensated by a subsequent decline further along the open-reading frame; however, none of these changes reached statistical significance (Fig. 2C). These fluctuations suggest that the changes are likely nonspecific and may arise stochastically.

**Figure 3.**
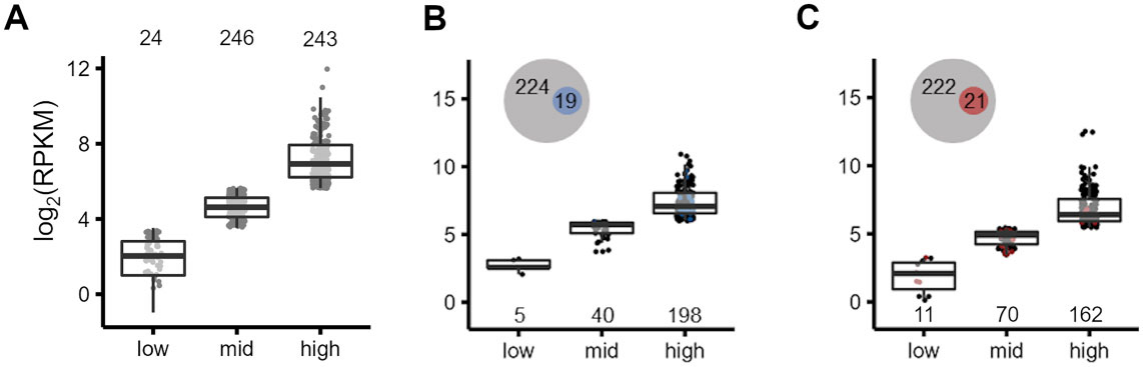
The m^6^A modification does not affect transcript expression levels under stress. (**A**) The expression levels [log_2_(RPKM)] in the control condition were split into three equally sized groups (low, mid, and high). Dark gray dots represent the transcripts identified as being methylated, depicted also as numbers above the plot. The 243 highly expressed and methylated transcripts in the control condition were split according to their expression levels under OS (**B**) or HS (**C**). The Venn diagrams in panels (B) and (C) show the number of transcripts that lose methylation under stress (larger circle) and the number of genes that remain methylated (smaller circle). m^6^A-seq (A–C) was performed on two independent biological replicates for each condition and both replicates were merged into one metaset. The numbers in the plots of panels (B) and (C) denote the corresponding number of transcripts in each category.

To investigate whether the decrease in m^6^A modification levels under stress caused a shift in gene expression, we assessed expression level of transcripts from the RNA-seq data. Based on the expression in the control condition, we divided the transcripts into three equal groups, i.e. low, medium, and high expression. The vast majority of the methylated transcripts in the control condition were either in the medium (246 of 513 transcripts) or in the highly expressed (243 of 513 transcripts) groups (Fig. 3A). Under OS, the m^6^A transcripts scored in the mid-level expression group, whereas under HS, the m^6^A transcripts scored in the lowly expressed group (Supplementary Fig. S5A and B). This pattern is distinct from that of the control. We also assessed the expression levels of the methylated transcripts that are highly expressed in the control condition (i.e. 243 transcripts). The majority of these transcripts remained highly expressed under OS and HS (i.e. 198 of 243 in OS, and 162 of 243 in HS), even though most of them lost m^6^A (Fig. 3B and C). This result suggests that the methylation does not contribute to the high expression level of the transcripts. The small set of transcripts preserving their methylation status under stress showed no specific expression patterns and were distributed amongst the three expression subgroups (colored dots Fig. 3B and C). The low-level expressed transcripts that were methylated under OS or HS were also low-level expressed in the control condition (Supplementary Fig. S5C and D), indicating that methylation does not affect the levels of gene expression in response to stress.

To further support the notion that the observed m^6^A-mRNA methylation does not mediate the response to stress, we compared the growth of four strains, *yafS*-KO, WT, *yafS*-KO, and WT, both of each with overexpressed *yafS*, under permissive growth conditions and when subjected to OS. Following OS, the growth of all strains, including WT, plateaued earlier than in the unstressed cells (Supplementary Fig. S1B and C). The YafS overexpression did not improve the growth—the growth curve of cells expressing plasmid-borne *yafS* did not differ from that of the WT or *yafS*-KO strains (Supplementary Fig. S1C). Furthermore, the similarity of the growth curves of WT and *yafS*-KO in response to OS implies no obvious role of *yafS* in oxidative stress response. Overall, our results suggest that m^6^A methylation in *E. coli* mRNAs is unlikely mediated by an enzyme-catalyzed mechanism that is sensitive to stress.

### m^6^A modification is likely randomly installed on mRNA in *E. coli*

**Figure 4.**
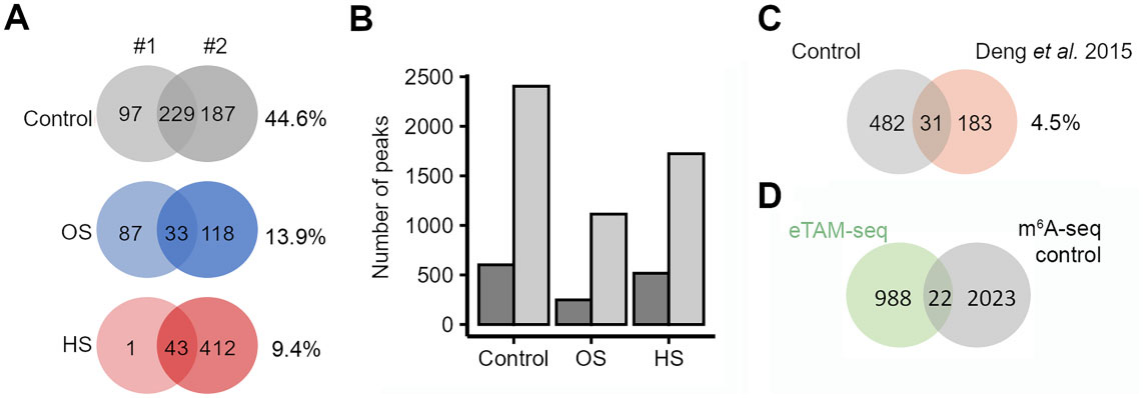
The m^6^A modification on *E. coli* mRNA is stochastic. (**A**) The overlap of transcripts methylated in each biological replicate (#1 and #2) in the control, OS, or HS. The percent shared genes was calculated as $\% \;{\rm shared}\,{\rm genes} = \;\frac{{{\rm shared}\,{\rm genes}}}{{{\rm total}\,{\rm genes}\;}}\; \times 100$. (**B**) The number of m^6^A peaks identified within the proposed m^6^A motifs UGGCAG, GCCAU, and CAGAUC (dark gray) compared to the total number of methylation peaks identified regardless of being within an m^6^A motif (light gray). (**C**) Overlap of transcripts identified as methylated in this study compared to those previously identified with a similar antibody-based approach in [27]. The percentage denotes the overlap. In panels (A) and (C), transcript identities are considered in the comparison, i.e. transcripts with at least one m^6^A peak irrespective of its position along the transcript. (**D**) Overlap of the m^6^A peaks in m^6^A-seq and persistent A signals in eTAM-seq. Note that persistent A signals in eTAM-seq might be either m^6^A or structured adenosines resistant to conversion. Both sequencing sets were performed in two independent biological replicates. All identified m^6^A peaks in the two independent biological replicates of m^6^A-seq, i.e. without further filtering for presence in motifs, were merged into a metaset. Some m^6^A peaks contained 2 or 3 signals from eTAM-seq; i.e. 22 overlapping m^6^A peaks from the m^6^A-seq contain 26 A signals from eTAM-seq.

Our observations do not support m^6^A modification of bacterial mRNA by a mechanism of direct enzyme recognition, but rather raised the possibility of a random m^6^A installing or incorporating. Indeed, we detected low overlap in the identified m^6^A-methylated sites among biological replicates in our m^6^A-seq datasets (i.e. 44.6%, 13.9%, and 9.4% shared transcripts in control sample, OS, and HS, respectively; Fig. 4A). Thus, considering the limitations of an antibody-based sequencing approach (e.g. m^6^A-seq), this lack of reproducibility contrasts the much higher reproducibility of m^6^A peaks of eukaryotic samples between different studies and biological replicates, even using the same sequencing approach (up to 60%) [50].

Typically, in the m^6^A-seq, which utilizes antibody-dependent recognition of the m^6^A modification, only m^6^A peaks that fall in consensus methylation motifs are considered as truly positive. However, after m^6^A peak calling, we noticed a large loss of m^6^A sites when considering only peaks within the described consensus motifs (UGGCAG, GCCAU, and CAGAUC) [27]. Therefore, we compared the number of m^6^A peaks within motifs and the total number of identified m^6^A peaks (Fig. 4B). The much higher number of total m^6^A peaks indicates that these peaks were largely not associated with the proposed consensus sequences [27], which further supports the notion for a lack of enzyme-mediated recognition and m^6^A installation.

We next compared our set of methylated genes from the control condition to a previous set of methylated transcripts identified in *E. coli* using the same sequencing approach [27]). Interestingly, we observed only a small overlap of methylated genes between the two datasets, supporting the idea that m^6^A modification of *E. coli* mRNAs is likely random (Fig. 4C).

The antibody-based m^6^A-seq lacks nucleotide precision in determination of m^6^A sites and has several limits in robustly detecting methylated sites [50]. We thus, considered eTAM-seq, allowing the identification of m^6^A positions with nucleotide precision [22]. Because with bacterial samples, FTO treatment is not possible (see “Materials and methods” section), persistent A signals in eTAM-seq might be either m^6^A or structured adenosines. We compared the 1014 A sites with over 50% persistent A signals in eTAM-seq to the peaks in m^6^A-seq, reasoning that a strong overlap would confirm these as genuine m^6^A sites. For comparison, we considered all m^6^A peaks from the m^6^A-seq without filtering for enrichment in specific motifs. m^6^A-seq and eTAM-seq revealed a poor correlation, with only 22 m^6^A-seq peaks encompassing eTAM-seq signals (Fig. 4D), suggesting that the persistent A sites detected by eTAM-seq are likely structured adenosines rather than m^6^A modifications. Together, the lack of overlap among the methylated transcripts between different sequencing methods and biological replicates suggests rather a random and unspecific incorporation of m^6^A in bacterial mRNAs.

## Conclusions

In this study, we investigated the mechanism and function that underlie the m^6^A modification on prokaryotic mRNA, using *E. coli* as an example. Based on the mechanism of m^6^A methylation in eukaryotes [16], we assumed a direct enzymatic recognition and methylation by methyltransferases that would specifically recognize unique sequence positions in bacterial mRNAs. In the entire *E. coli* genome, we identified four candidates with yet uncharacterized function but bearing putative methyltransferase domains. Despite the decrease of m^6^A modification levels following the knockout of each, the *in vitro* methylation assays reveal no activity, thus disregarding them as m^6^A-mRNA methyltransferases. Additionally, while we observed changes of m^6^A levels in cells exposed to OS and HS, which corroborates previous findings [27], different sets of transcripts were methylated under stress that do not overlap with the modified transcripts detected in cells without stress. These results, combined, suggest that unlike in eukaryotes, the m^6^A incorporation in bacterial mRNA lacks a direct involvement of enzymes and shows no perceivable effect in cells with or without stress.

The lack of reproducibility between the m^6^A positions and even the identity of the modified transcripts among biological replicates, along with the lack of overlap between two different sequencing methods, further supports the notion that the m^6^A modification occurrence in bacterial mRNA is rather random. While the presence of m^6^A in the CDS alters translational decoding of the m^6^A modified codons [61–63], the randomness of the m^6^A distribution in bacterial mRNAs suggests that such changes of the decoding efficacy may not be biologically meaningful. Consistent with this notion, increasing evidence suggests that, despite the clear indications for functional benefit of some m^6^A sites in eukaryotes [2, 3, 9, 64–66], the majority of those in the 5′ UTR and in protein coding regions (i.e. up to 80%) are also nonfunctional and not subject to the purifying evolutionary selection [2, 33, 67, 68].

While we could not identify a dedicated methyltransferase that installs site-specific m^6^A in *E. coli* mRNA, it remains possible that the methylation is incorporated by other mechanisms that are not conserved with those in eukaryotes. One possibility would be by direct incorporation of already methylated adenine or adenosine nucleosides or nucleotides, which could be randomly incorporated into mRNA during transcription. A study has identified a metabolic enzyme that deaminates *N*^6^-methyladenosine to generate inosine in *B. subtilis* [67], which can then be converted to inosine triphosphate and randomly incorporated into mRNA during transcription. Notably, while we were unable to identify methylated adenosine, AMP, or ATP by incubation with the purified YafE or YafS, this negative result does not entirely invalidate the hypothesis of random incorporation of already modified m^6^A. Indeed, we cannot exclude the possibility that methylated adenosine, AMP, or ATP could be generated by other metabolic enzymes, and consequently randomly incorporated into mRNA during transcription. Modified purines have been found to be incorporated into bacterial DNA [69]; however, there is no evidence of modified purines that can be incorporated into bacterial RNA. Alternatively, m^6^A monophosphates can be generated as a degradation product from rRNA, where abundant m^6^A nucleotides reside, or from tRNA^Val^(UAC), which is the single *E. coli* tRNA with site-specific m^6^A (position 37). The released m^6^A monophosphates could be converted to the triphosphate form and incorporated into mRNA during transcription. A recent study describes how a CRISPR-associated bacterial Cad1 enzyme converts ATP to ITP in stress, suggesting that chemical modifications of NTPs are possible [70]. This finding also raises the possibility that, at some point in the evolutionary trajectory of *E. coli*, other enzymes may have evolved to convert ATP to m^6^ATP.

In summary, our results show that m^6^A methylation in bacterial mRNA is unlikely mediated by a direct and site-specific enzymatic mechanism as in eukaryotes. Rather, we suggest that m^6^A methylation in bacterial mRNA is random and likely a relic of nucleotide salvage pathways that generate the modified adenosine moiety, which is then converted to an m^6^A triphosphate and subsequently incorporated into bacterial mRNA. This random m^6^A modification on bacterial mRNAs is unlikely to confer functional benefit.

## Supplementary Material

gkaf425_Supplemental_File

## Data Availability

RNA-seq, m^6^A-seq, and eTAM-seq are deposited in the Gene Expression Omnibus (GEO) under the accession numbers GSE206340 and GSE281945, respectively.
