# Supplementary material for: m6A modification is incorporated into bacterial mRNA without specific functional benefit
Source: Nucleic Acids Res. 2025 May 22;53(10):gkaf425. doi: 10.1093/nar/gkaf425 (PMC12096079; doi:10.1093/nar/gkaf425)
Supplement: gkaf425_Supplemental_File [file gkaf425_supplemental_file.pdf]

Supplementary information

m^6A methylation is incorporated randomly into bacterial mRNA without specific functional benefit

Klara Szydlo[1]†, Leonardo Santos[1]†, Thomas W. Christian[2], Sunita Maharjan[2], Amir Dorsey[2], Isao Masuda[2], Jingxuan Jia[3,4], Yuan Wu[3,4], Weixin Tang[3,4], Ya-Ming Hou[2*], and Zoya Ignatova[1]*

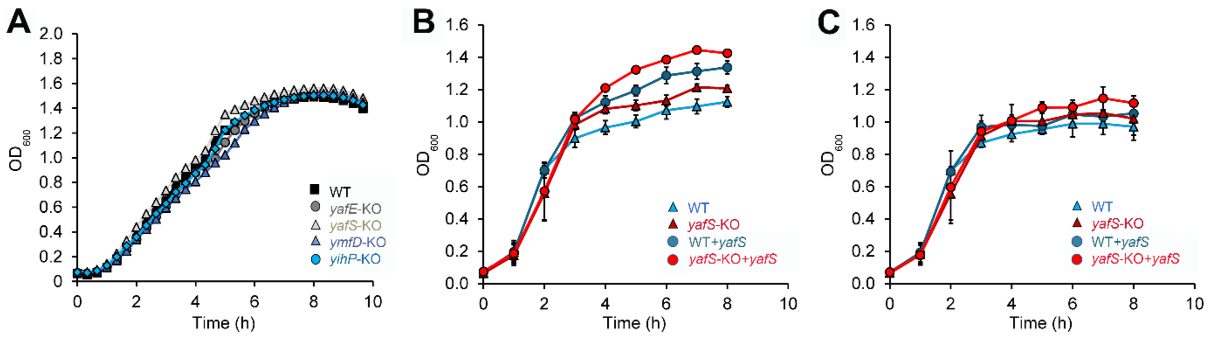

Supplementary Figure S1. Growth curve analysis of different *E. coli* strains. (A) KO of the four putative m^6A methyl transferases did not affect *E. coli* growth. WT, wildtype *E. coli* MG1655 strain. (B) Growth curves of WT, *yafS*-KO, and over-expression of a plasmid-borne *yafS* in WT and in *yafS*-KO. Overexpression of the plasmid-borne *yafS* similarly enhances growth of the host strain in the stationary phase with no discernible differences between WT and *yafS*-KO. (C) Overexpression of a plasmid-borne *yafS* in *yafS*-WT or *yafS*-KO following OS, i.e. in presence of 1 mM paraquat does not change the growth profile. Color codes are the same as in (B). Data (A-C) are means ± SD (n=3 independent biological replicates).

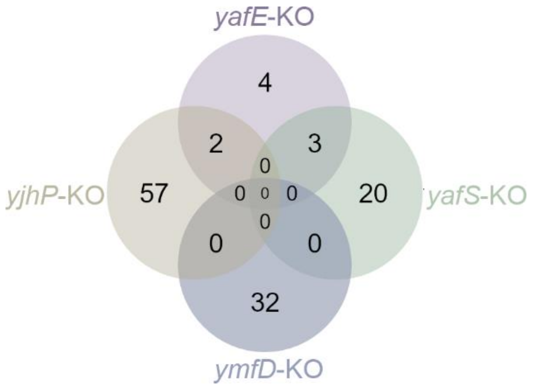

Supplementary Figure S2. No overlap between methylated transcripts in the KO strains. A comparison between the identity of the transcripts with at least one m^{6}A peak in the different KO strains.

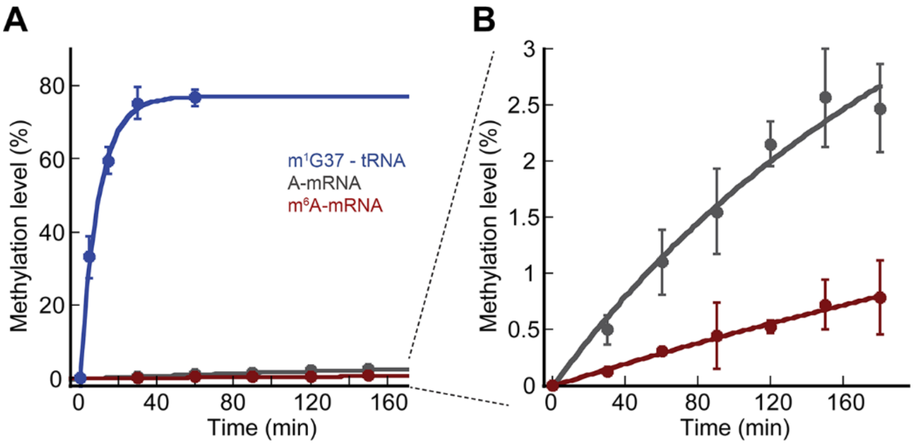

Supplementary Figure S3. Methylation activity of a whole cell lysate from *E. coli* MG1655. (A) The whole cell lysate displayed high N^1 methylation activity on tRNA and synthesized m^1G37 to 80% within 60 min, but had no significant m^6A activity on A-mRNA (gray, red). (B) Enlarged view of the methylation activity of the whole cell lysate on A-mRNA (gray) or the m^6A-mRNA (red). The methylation on the A-mRNA (gray) was similar to that of the already methylated m^6A-mRNA (red) substrate. Data are means ± SD (n=3 independent biological replicates). Experimental data points were fit to a single exponential equation based on searches for the best-fit.

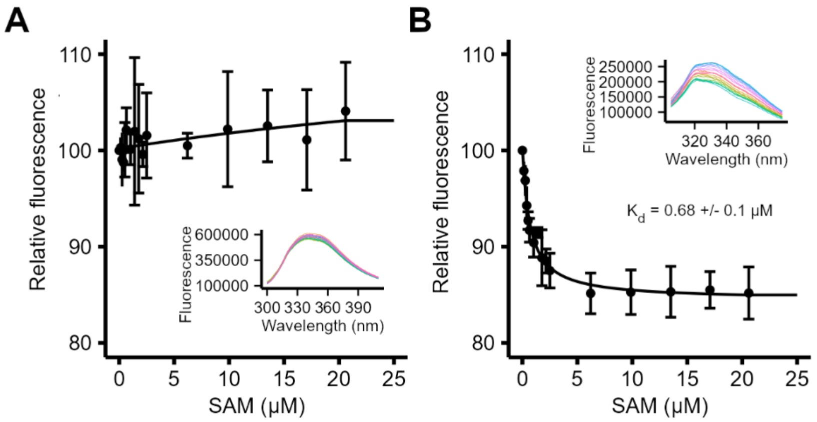

Supplementary Figure S4. Determination of the equilibrium binding of SAM to purified YafE and YafS in the absence of an mRNA substrate. (A, B) Quenching of the intrinsic tryptophan fluorescence of YafE (A) and YafS (B) following titration with different SAM concentrations. Single emission scans of the titration series are shown in the insets. In (B), the amplitude of the change was 15%. Curve fit resulted in a K_d = 0.68 +/- 0.1 µM. Data are means ± SD (n=3 independent replicates).

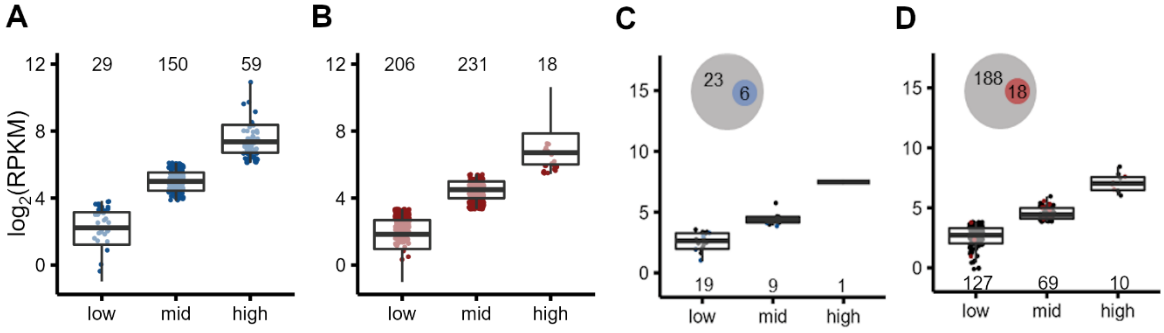

Supplementary Figure S5. The m^{6}A modification does not affect transcript expression levels under stress. The expressed transcripts (log_2(RPKM)) under OS (A) or HS (B) were split into three equally sized expression subgroups of expression level (low, mid, high). Dark colored dots represent the methylated transcripts within each subgroup, depicted also as numbers above the plot. (C, D) Expression levels of the transcripts that were lowly expressed and methylated under OS (29 transcripts, C) or HS (206 transcripts, D) and further split according to their expression levels in the control condition. The Venn diagrams display the number of transcripts that lose methylation (grey) and transcripts that keep their methylation status (blue circle for OS (C) and red circle for HS (D), respectively) relative to the control condition. m^{6}A-seq (A-C) were performed on two independent biological replicates for each condition and both replicates were merged into one meta set.

Supplementary Table 1. High confidence hits of the homology search of proteins with methylase domains in the *E. coli* proteome. In total six genes have uncharacterized functions, two of which have unlikely function as methyltransferases on nucleic acids (e.g. *speE*, a methyl transferase in polyamine biosynthesis, and *ynbC*, a putative esterase) were excluded. The remaining four putative methyltransferases selected for further investigation are highlighted in bold.

Gene name	Gene Code	Transcript	EggNOG	Description
rsmA	b0051	AAC73162	COG0030	16S rRNA m(6)A1518, m(6)A1519 dimethyltransferase, SAM-dependent
rlmD	b2785	AAC75827	COG0030<br>COG0500<br>COG2226	23S rRNA m(5)U1939 methyltransferase, SAM-dependent
cfa	b1661	AAC74733	COG0030<br>COG0500<br>COG2226	cyclopropane fatty acyl phospholipid synthase, SAM-dependent
bioC	b0777	AAC73864	COG0030<br>COG0500<br>COG2226	malonyl-ACP O-methyltransferase, SAM-dependent
trmN	b2575	AAC75628	COG0030<br>COG0286<br>COG0500<br>COG2226	tRNA1(Val) (adenine(37)-N6)-methyltransferase
smtA	b0921	AAC74007	COG0030<br>COG0500<br>COG2226	putative S-adenosyl-L-methionine-dependent methyltransferase
ubiE	b3833	AAT48227	COG0030<br>COG0500<br>COG2226	bifunctional 2-octaprenyl-6-methoxy-1,4-benzoquinone methylase/ S-adenosylmethionine:2-DMK methyltransferase
prmC	b1212	AAC74296	COG0030<br>COG0286<br>COG0500<br>COG2226	RF-1 and RF-2 N5-glutamine methyltransferase
tam	b1519	AAC74592	COG0030<br>COG0500<br>COG2226	trans-aconitate methyltransferase
rlmA	b1822	AAC74892	COG0030<br>COG0500<br>COG2226	rRNA m(1)G745 methyltransferase, SAM-dependent
cmoA	b1870	AAC74940	COG0030<br>COG0500<br>COG2226	carboxy-SAM synthase
prmA	b3259	AAC76291	COG0030<br>COG0500<br>COG2189<br>COG2226	methyltransferase for 50S ribosomal subunit protein L11
ubiG	b2232	AAC75292	COG0030<br>COG0500<br>COG2226	bifunctional 3-demethylubiquinone-9 3-methyltransferase/ 2-octaprenyl-6-hydroxy phenol methylase
rsmB	b3289	AAC76314	COG0030<br>COG2226	16S rRNA m(5)C967 methyltransferase, SAM-dependent
rsmC	b4371	AAC77324	COG0030<br>COG0286<br>COG0500<br>COG2226	16S rRNA m(2)G1207 methyltransferase, SAM-dependent
pcm	b2743	AAC75785	COG0030<br>COG0500<br>COG2226	L-isoaspartate protein carboxylmethyltransferase type II
rsmD	b3465	AAC76490	COG0030	16S rRNA m(2)G966 methyltransferase, SAM-dependent
lgoD	b4358	AAC77314	COG0030<br>COG0500<br>COG2226	L-galactonate oxidoreductase; L-gulonate oxidoreductase
hsdM	b4349	AAC77305	COG0286	DNA methyltransferase M
rlmL	b0948	AAC74034	COG0286<br>COG0338<br>COG0500<br>COG2189<br>COG2226	23S rRNA m(2)G2445 and m(7)G2069 methyltransferases, SAM-dependent
prmB	b2330	AAC75390	COG0286<br>COG0500	N5-glutamine methyltransferase

			COG2189<br>COG2226	
rlmG	b3084	AAC76119	COG0286<br>COG0500<br>COG2226	23S rRNA m(2)G1835 methyltransferase, SAM-dependent
rlmI	b0967	AAC74053	COG0286<br>COG0500<br>COG2226	23S rRNA m(5)C1962 methyltransferase, SAM-dependent
dam	b3387	AAC76412	COG0338<br>COG3392	DNA adenine methyltransferase
yhdJ	b3262	AAC76294	COG0338<br>COG0500<br>COG2189<br>COG3392	DNA adenine methyltransferase, SAM-dependent
yjhP	**b4306**	**AAC77262**	**COG0500<br>COG2226**	**putative methyltransferase**
cmoB	b1871	AAC74941	COG0500<br>COG2226	tRNA (cmo5U34)-carboxymethyltransferase, carboxy-SAM-dependent
tehB	b1430	AAC74512	COG0500<br>COG2226	tellurite, selenium methyltransferase, SAM-dependent; tellurite, selenium resistance protein
fabB	b2323	AAC75383	COG0500<br>COG2226	3-oxoacyl-[acyl-carrier-protein] synthase I
yafE	**b0210**	**AAC73315**	**COG0500<br>COG2226**	**putative S-adenosyl-L-methionine-dependent methyltransferase**
speE	b0121	AAC73232	COG0500<br>COG2226	spermidine synthase (putrescine aminopropyltransferase)
fabF	b1095	AAC74179	COG0500<br>COG2226	3-oxoacyl-[acyl-carrier-protein] synthase II
ymfD	**b1137**	**AAC74221**	**COG0500<br>COG2226**	**e14 prophage; putative SAM-dependent methyltransferase**
ynbC	b1410	AAC74492	COG0500<br>COG2226	putative esterase
cheR	b1884	AAC74954	COG0500<br>COG2226	chemotaxis regulator, protein-glutamate methyltransferase
rsmF	b1835	AAC74905	COG0500<br>COG2226	16S rRNA m(5)C1407 methyltransferase, SAM-dependent
rlmE	b3179	AAC76211	COG0500<br>COG2226	23S rRNA U2552 2'-O-ribose methyltransferase, SAM-dependent
rlmC	b0859	AAC73946	COG0500<br>COG2226	23S rRNA m(5)U747 methyltransferase, SAM-dependent
trmA	b3965	AAC76947	COG0500<br>COG2226	tRNA m(5)U54 methyltransferase, SAM-dependent; tmRNA m(5)U341 methyltransferase
trmI	b2960	AAC75997	COG0500	tRNA m(7)G46 methyltransferase, SAM-dependent
yafS	**b0213**	**AAC73318**	**COG0500<br>COG2226**	**putative S-adenosyl-L-methionine-dependent methyltransferase**
gpsA	b3608	AAC76632	COG0500<br>COG2226	glycerol-3-phosphate dehydrogenase (NAD+)
yahK	b0325	AAC73428	COG0500	broad specificity NADPH-dependent aldehyde reductase, Zn-containing
rlmJ	b3499	AAC76524	COG2189	description:16S rRNA m(2)G1516 methyltransferase, SAM-dependent
tdh	b3616	AAC76640	COG2226	L-threonine 3-dehydrogenase, NAD(P)-binding
rsmH	b0082	AAC73193	COG2226	16S rRNA m(4)C1402 methyltransferase, SAM-dependent
adhP	b1478	AAC74551	COG2226	ethanol-active dehydrogenase/acetaldehyde-active reductase
paaH	b1395	AAC74477	COG2226	3-hydroxyadipyl-CoA dehydrogenase, NAD+-dependent
